# Evaluation of the immunogenicity and safety of different doses and formulations of a broad spectrum influenza vaccine (FLU-v) developed by SEEK: study protocol for a single-center, randomized, double-blind and placebo-controlled clinical phase IIb trial

**DOI:** 10.1186/s12879-017-2341-9

**Published:** 2017-04-04

**Authors:** Eva van Doorn, Olga Pleguezuelos, Heng Liu, Ana Fernandez, Robin Bannister, Gregory Stoloff, Fredrik Oftung, Stephen Norley, Anke Huckriede, Henderik W. Frijlink, Eelko Hak

**Affiliations:** 1 University of Groningen, Unit of PharmacoTherapy- Epidemiology & -Economics, Antonius Deusinglaan, 9713 AV, Groningen, The Netherlands; 2 SEEK, Central Point, 45 Beech Street, London, EC2Y 8AD UK; 3 Norwegian Institute of Public Health, Department of Infectious Disease Immunology, Oslo, Norway; 4 Robert Koch Institute, Berlin, Germany; 5 University Medical Center Groningen, Medical Microbiology, Groningen, The Netherlands; 6 Department of Pharmaceutical Technology and Biopharmacy, University of Groningen, Groningen, The Netherlands

**Keywords:** Broadly protection, Clinical trial, CMI, FLU-v, Influenza, Universal, Vaccine

## Abstract

**Background:**

Current influenza vaccines, based on antibodies against surface antigens, are unable to provide protection against newly emerging virus strains which differ from the vaccine strains. Therefore the population has to be re-vaccinated annually. It is thus important to develop vaccines which induce protective immunity to a broad spectrum of influenza viruses. This trial is designed to evaluate the immunogenicity and safety of FLU-v, a vaccine composed of four synthetic peptides with conserved epitopes from influenza A and B strains expected to elicit both cell mediated immunity (CMI) and humoral immunity providing protection against a broad spectrum of influenza viruses.

**Methods:**

In a single-center, randomized, double-blind and placebo-controlled phase IIb trial, 222 healthy volunteers aged 18–60 years will be randomized (2:2:1:1) to receive two injections of a suspension of 500 μg FLU-v in saline (arm 1), one dose of emulsified 500 μg FLU-v in Montanide ISA-51 and water for injection (WFI) followed by one saline dose (arm 2), two saline doses (arm 3), or one dose of Montanide ISA-51 and WFI emulsion followed by one saline dose (arm 4). All injections will be given subcutaneously. Primary endpoints are safety and FLU-v induced CMI, evaluated by cytokine production by antigen specific T cell populations (flow-cytometry and ELISA). Secondary outcomes are measurements of antibody responses (ELISA and multiplex), whereas exploratory outcomes include clinical efficacy and additional CMI assays (ELISpot) to show cross-reactivity.

**Discussion:**

Broadly protective influenza vaccines able to provide protection against multiple strains of influenza are urgently needed. FLU-v is a promising vaccine which has shown to trigger the cell-mediated immune response. The dosages and formulations tested in this current trial are also estimated to induce antibody response. Therefore, both cellular and humoral immune responses will be evaluated.

**Trial registration:**

EudraCT number 2015–001932-38; retrospectively registered clinicaltrials.gov NCT02962908 (November 7th 2016).

## Background

Influenza virus is an important respiratory pathogen that causes annual epidemics and more seldom global pandemics [1, 2]. Influenza disease is generally characterized by an acute onset of fever, muscle and joint pain, and respiratory symptoms (e.g. coughing, sneezing, running nose, sore throat) [1, 3, 4]. The symptoms are in most cases self-limiting. However, influenza is associated with high mortality and morbidity rates, especially in the elderly, children younger than two years of age, pregnant women, and individuals suffering from chronic diseases or weakened immune system [3, 4]. Typical complications from influenza infection which may result in hospitalizations and deaths include pneumonia, caused by the primary influenza infection itself or secondary bacterial infections, and the exacerbation of chronic illness (i.e. pulmonary or cardiovascular diseases) [1, 4]. Depending on the virulence of the strains circulating during a particular influenza season, three to five million cases of severe illness and about 250.000 to 500.000 deaths can occur worldwide [3, 5]. Influenza also has a significant financial impact due to medical treatment, hospitalizations and work absenteeism [6].

The primary measure for preventing influenza disease is vaccination [3, 4]. For over 70 years, public health programs have included influenza vaccines that are able to neutralize the virus by eliciting antibodies against the surface antigens hemagglutinin (HA) and neuraminidase (NA) [7]. HA is responsible for the entry of the virus into the host cells through the attachment of the viral antigen to the sialic-acid-containing receptors on the host cell surface, whereas NA facilitates budding of newly formed viral particles and viral movement along the respiratory tract to the target cell by cleaving sialic acid [8, 9]. HA and NA exhibit a high variability due to continuous changes of the viral genome through antigenic drift or shift. Antigenic drift is caused by point mutations occurring during viral replication which leads to minor amino acid changes resulting in new circulating virus strains each year [1, 10]. Antigenic shift is an abrupt and major change of influenza virus due to genetic re-assortment between human and other animal influenza viruses when a host is co-infected by both viruses [1, 10]. Occasionally, these antigenic shifts result in new influenza A subtypes that have not been circulating in the human population before causing pandemics [1, 2].

Current influenza vaccines are safe and effective. However, the efficacy and effectiveness is limited. Due to ongoing antigenic drift, the influenza vaccine has to be updated annually and the population has to be re-vaccinated. Efficacy of the vaccine is mainly dependent on the degree of similarity between the vaccine and the circulating strains [11]. When having a good match, vaccination can reduce the risk of illness by 50–60% among the overall population [11]. However, a mismatch situation can reduce the vaccine protective efficacy drastically leading to more influenza related hospitalizations and deaths. A mismatch occurs due the fact that manufacturing of the vaccine must start 6 months in advance of the influenza season. The World Health Organization can only predict based on epidemiological studies which influenza strains are likely to circulate. However, the prediction is not always accurate or new strains can emerge that could not have been predicted. Thus, there is a need for influenza vaccines that protect the population against a broad spectrum of influenza viruses, including both seasonal and pandemic strains.

FLU-v was developed by SEEK (UK) to provide broad spectrum protection against influenza. Initially FLU-v will be administrated in conjunction with the annual influenza vaccine to broaden the protection and protect subjects in case of vaccine mismatch. FLU-v contains a sterile equimolar mixture of four synthetic polypeptides that cover highly conserved regions (regions that do not undergo antigenic changes) from the nucleoprotein (NP), matrix 1 (M1) and matrix 2 (M2) proteins of both the human and animal influenza A and B virus strains. NP, M1 and M2 are internal proteins which play an important role in the stimulation of T-cell responses during influenza infection [12]. Based on previous clinical studies, FLU-v primarily triggers cell-mediated immune responses [13]; however the dosages and formulations to be tested in this current trial are also estimated to induce antibody responses. These cellular and humoral responses are expected to mediate cross-protection against different influenza viruses. In this phase IIb trial the immunogenicity and safety of FLU-v will be evaluated in healthy volunteers aged 18 to 60 years. Immune responses to two different treatment options, one dose of adjuvanted FLU-v or two doses of non-adjuvanted FLU-v, will be assessed. Immunogenicity will be evaluated by measurement of influenza-specific cell-mediated immunity (CMI) and humoral responses. Cross-reactivity will be studied by ELISpot. Safety will be evaluated based on the reported adverse events (AEs) and serious adverse events (SAEs) during the study period. In addition, clinical efficacy will be evaluated as an exploratory endpoint by assessing the number of reverse transcriptase polymerase chain reaction (RT-PCR) confirmed influenza cases and the severity of disease symptoms.

## Method/design

### Study design

**Fig. 1:**
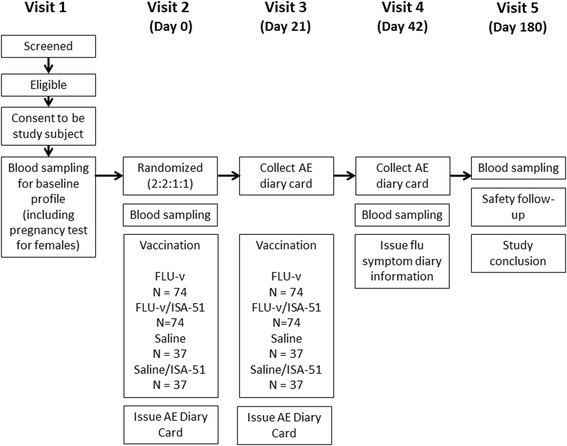
Flow chart of the study’s design

The immunogenicity and safety of FLU- v will be evaluated in a single-center, randomized, double-blinded and placebo-controlled trial. The study will be conducted at the Isala Clinics (Zwolle, The Netherlands). A total of 222 healthy volunteers aged 18 to 60 years will be recruited and followed for up to 6 months from screening to study conclusion. Individuals volunteering for the trial will be screened for their eligibility before enrollment. Written consent will be obtained during the screening visit (Figure 1). Eligible subjects will be randomized to receive one of four treatments; (1) two doses of FLU-v as a suspension in saline, (2) one dose of FLU-v as an emulsion in Montanide ISA-51 and water for injection (WFI) followed by one dose of saline, (3) two doses of saline, or (4) one dose of WFI and Montanide ISA-51 emulsion, followed by one dose of saline. All administrations will be given subcutaneously (s.c.) 21 ± 3 days apart. Blood samples will be taken from all subjects on day 0 (before the first administration), day 42 (21 days after the second administration) and day 180 (159 days after the second administration) for the evaluation of short term and long term vaccine-specific cellular and humoral immune responses. AE questionnaires will be issued on day 0 and 21 to subjects to follow solicited AEs. The questionnaires will need to be completed by the subjects and returned on day 21 and day 42. The incidence and nature of unsolicited AEs and SAEs will be followed during the whole study period. In addition, during the influenza season 2016–2017 subjects will need to record influenza symptoms daily by means of a web-based questionnaire. Subjects will receive a daily reminder to complete the questionnaire. Subjects will be instructed to record influenza symptoms and when subject experiences a sudden onset (within 24 h) of at least one respiratory (cough, sore throat, shortness of breath, runny nose, stuffy nose, sneezing and earache) and one systemic symptom (fever, malaise, headache and myalgia (muscle and joint pain)), nasal and tonsil samples will be taken within three days from contacting the subject or within four days of the onset of symptoms, whatever time is shorter. Confirmation of influenza infection will be performed by RT-PCR assay.

### Participants

Healthy males and females between the ages of 18 and 60 years are eligible for the study (Table 1). Pregnant or breast-feeding women, individuals who have received an influenza vaccine or have experienced influenza-like-illness within the 6 months prior to the study, those who are receiving medication or treatment that may affect the evaluation of their immune responses and those who have a history of chronic disease and/or immune system disorder will be excluded from trial participation (Table 1). Women of childbearing potential and men must agree to practice adequate contraception throughout the study treatment (up to day 51 (for female) and day 111 (for males)). Furthermore, individuals should be able to understand and comply with the planned study procedures and provide a signed informed consent form after receiving a detailed explanation of the study protocol prior to any of the study procedures. In case of uncertainty about the medical status of an individual regarding any of the exclusion criteria, the primary care physician will be consulted. Consultation of the primary care physician is included in the consent form and will only be related to medical information about the exclusion criteria.

**Table 1:** Trial inclusion and exclusion criteria trial subjects

Inclusion criteria	- Aged between 18 and 60 years
	- Healthy males and healthy non-pregnant females (as indicated by a negative blood pregnancy test during the screening visit)
	- Healthy as determined by vital signs (heart rate, blood pressure, oral temperature), blood chemistry test (electrolytes, renal/kidney function, liver function, C-reactive protein, complete blood count), medical history, general physical examination, self-reported illness and the clinical judgment of the investigator
	- Women of childbearing potential (not surgically sterile or postmenopausal for ≥1 year) and men must agree to practice adequate contraception (a combination of barrier and hormone methods for women and a condom for men) throughout the study treatment for at least up 30 days (to day 51 for females) and 90 days (to 111 for males) after the last vaccination
	- Able to understand and comply with planned study procedures
	- Provides signed informed consent form after receiving a detailed explanation of the study protocol prior to any study procedures
Exclusion criteria	- Known allergy to any of the components of the vaccine
	- History of severe reactions following immunization
	- Immune deficiency/disorder, whether due to genetic defect, immunodeficiency disease, or immunosuppressive therapy
	- Positive pregnancy test during the screening visit or who are breastfeeding
	- History of (reported by subjects): Acute disseminated encephalomyelitis (ADEM); Neoplastic disease – current or previous; Asthma or severe allergic disease; Bleeding disorders; Chronic hepatitis B and/or C infection; Chronic liver disease; Diabetes mellitus; Guillain-Barre syndrome; Human immunodeficiency virus (HIV); Rheumatoid arthritis or other autoimmune diseases; Severe renal disease; Transplant recipients; Unstable or progressive neurological disorders
	- Receipt of medicines/treatment that may affect the evaluation of immunogenicity such as; Oral or parenteral steroids, high-dose inhaled steroids (greater than 800 micrograms/day of beclomethasone dipropionate or equivalent) or other immunosuppressive or cytotoxic drugs (within the 6 months prior to vaccination in this study); Immunoglobulin or other blood products (within the 3 months prior to vaccination in this study); Experimental agent (vaccine, drug, biologic, device, blood product, or medication) within 1 month prior to vaccination in this study, or expects to receive an experimental agent during the study period; Influenza antiviral medication within the 4 weeks prior to the vaccination in this study.
	- Received any influenza vaccine within 6 months prior to vaccination in this study.
	- Influenza like-illness (a sudden onset of symptoms and at least one of the four systemic symptoms *fever, or feverishness, malaise, headache, myalgia* and at least one of the three respiratory symptoms *cough, sore throat, shortness of breath*) or acute respiratory infection (a sudden onset of symptoms and at least of the four respiratory symptoms *cough, sore throat, shortness of breath, coryza* and a clinician’s judgment that the illness is due to an infection) within 6 months prior to vaccination in this study. These symptoms must have stopped the subject from carrying out their normal daily activities such as attending work or school for a period of at least 3 days.
	- Acute illness, including an oral temperature greater than 38 °C, within 1 week before vaccination.
	- History of alcohol or drug abuse within the last 2 years deemed unsuitable for inclusion by the investigator.
	- Any abnormal haematology values and/or serum chemistries judged by the investigator as clinically significant.
	- Ineligible subject based on the judgement of the investigator.
	In case there is uncertainty about the participant’s medical status regarding any of the exclusion criteria mentioned, the participant’s primary care physician will be consulted. Consultation of the primary care physician will only take place after having received written approval from the participant, and will concern medical information about exclusion criteria only.

### Screening and baseline assessment

To ensure the health of subjects before entering the trial, a general physical examination will be performed during the screening visit and a blood sample will be collected for laboratory tests including hematology (e.g. hemoglobin, red blood cell count, platelet count) and serum chemistry (e.g. uric acid, creatinine, albumin, glucose). Female subjects will also be subjected to a blood pregnancy test. Subjects with a clinically significant abnormal test result will be excluded from the study. Additional information on demographics, self-reported medical and medication history, influenza vaccination history, and alcohol, drug and cigarette consumption will also be recorded. Each screened subject will receive a sequential screening number which the subjects will retain whether or not they are ultimately randomized to receive study treatment. Baseline serum samples (day 0) will be stored until the end of the trial to measure the hemagglutination inhibition (HI) antibody titers against the influenza virus strains circulating during the 2016–2017 season, since presence of such antibodies could have provided the subject a certain degree of protection against infection with those strains thus affecting the vaccine efficacy data.

### Interventions

Subjects eligible for the study will be randomized to one of the four treatment arms (ratio 2:2:1:1) as described in Table 2. Subjects in arm 1 will receive two doses of 500 microgram (μg) non-adjuvanted FLU-v in suspension in 0.5 ml of saline (made by adding 0.25 ml of 0.01 M HCl followed by 0.25 ml of 0.01 M NaOH). Subjects in arm 2 will receive one dose of adjuvanted 500 μg FLU-v as an emulsion made with 0.25 ml of Montanide ISA-51 and 0.25 ml of WFI, followed by one dose of 0.5 ml of saline. Two reference products will be used as controls for arm 1 and 2. In arm 3 subjects will receive two 0.5 ml doses of saline as a control for those receiving non-adjuvanted FLU-v (arm 1). Subjects in arm 4 will receive one dose of 0.5 ml emulsion prepared with 0.25 ml of WFI and 0.25 ml of Montanide ISA-51, followed by 0.5 ml of saline as a control for those receiving the adjuvanted emulsion of FLU-v (arm 2). All treatments will be given as a subcutaneous injection in the upper section of the arm with an interval of 21 ± 3 days between the two doses.

**Table 2:** Vaccination schedule

Arm	Administration 1 (day 0)	Administration 2 (day 21)
1	500 μg FLU-v in 0.5 ml saline^a^ (suspension)	500 μg FLU-v in 0.5 ml saline^a^ (suspension)
2	500 μg FLU-v in 0.25 ml WFI and 0.25 ml Montanide ISA-51 (emulsion)	0.5 ml saline (solution)
3	0.5 ml saline (solution)	0.5 ml saline (solution)
4	0.25 ml WFI and 0.25 ml Montanide ISA-51(emulsion)	0.5 ml saline (solution)

^a^Made by mixing 0.25 ml of 0.01 M HCl and 0.25 ml 0.01 sM NaOH

### Outcomes

#### Primary outcomes

The primary outcomes of the study are safety and Th1 CMI responses elicited by FLU-v. Safety evaluation will include the solicited AEs in all subjects until 21 days after the last administration of the study vaccine and the unsolicited AEs and SAEs in all subjects during the whole study period. The intensity and causality of all reported AEs will be assessed by the investigator (Table 3). CMI at days 0, 42 and 180 will be evaluated by intracellular cytokine staining (ICS) analysis after 24 h of in vitro stimulation of peripheral blood mononuclear cells (PBMC) with vaccine antigen. This analysis will inform about the cytokine pattern (interleukin (IL) 2, interferon gamma (IFN-γ) and tumour necrosis factor alpha (TNFα)), phenotypic markers (clusters of differentiation 3 (CD-3), CD-4 and CD-8) and cytotoxic potential (CD107a) of antigen specific T cell populations activated by the vaccine. Vaccine specific Th1 responses will also be evaluated by IFN-γ enzyme-linked immunosorbent assay (ELISA) in supernatants from PBMC cultures exposed to the same antigens.

**Table 3:** Adverse events classification

Adverse event intensity	
Mild	An event that is easily tolerated by the subject, causing minimal discomfort and not interfering with everyday activities
Moderate	An event that causes sufficient discomfort as to interfere with normal everyday activities
Severe	An event that prevents normal everyday activities
Serious	Any untoward medical occurrence that: o Results in death o Is life threating (an event in which the subject was at risk of death at the time of the event) o Requires hospitalization or prolongation of an existing hospitalization o Results in disability or permanent damage as defined as a substantial disruption of a person’s ability to conduct normal life functions o Is a congenital anomaly/birth defect o Other adverse events that may jeopardize the subject or may require medical or surgical intervention to prevent one of the other outcomes
Adverse event causality	
Unrelated	Where an event is not considered related to the investigational medicinal product
Unlikely	Although the relationship to investigational medicinal product cannot be completely ruled out, the nature of the event, the underlying disease, concomitant medication or temporal relationship make other explanations more likely
Possibly related	The temporal relationship and the absence of a more likely explanation suggest the event could be related to the investigational medicinal product
Probably related	The known effects of the investigational medicinal product or its therapeutic class, or based on challenge testing, suggest the investigational medicinal product is the most likely cause
Definitely related	This category applies to those AEs that are clearly a consequence of administration of the drug. It is likely that such events will be widely documented and generally accepted as having association with the study medication.

#### Secondary outcomes

Secondary outcomes are the evaluation of antibody responses specific for FLU-v at days 0, 42 and 180 analyzed by total Ig ELISA, followed by isotyping the antibody response using a multiplex approach in samples with high FLU-v specific Ig titer.

#### Exploratory outcome

Exploratory outcomes are (1) FLU-v cross-reactive immunity evaluated with additional CMI assays such as dual ELISpot for IFN-γ and granzyme B, and (2) clinical efficacy of the FLU-v vaccine. Clinical efficacy will be evaluated as the reduction of the incidence of RT-PCR confirmed influenza A and/or B infections and the reduction in the symptom scores in subjects with confirmed influenza infections during the 2016–2017 influenza season. The relationship between efficacy and the immune response will be explored if possible. Moreover, the effect of previous influenza vaccination on the immunogenicity of FLU-v will be assessed in a post-hoc exploratory analysis with data stratified by the length of time between the last influenza vaccination and the current study treatment.

### Sample size calculation

The sample size is determined on the basis of influenza specific IFN-γ responses since this is one of the most important CMI markers for protection against influenza [14, 15]. Based on a previous phase I trial with FLU-v it is expected that at least a two-fold increase in the IFN-γ response measured with ELISA will be observed in the FLU-v non-adjuvanted arm (arm 1) when compared to the non-adjuvanted placebo (arm 3) [13]. In comparison, a five-fold increase in the IFN-γ response is expected to be observed in the adjuvanted-FLU-v arm (arm 2) when compared to the adjuvanted-placebo (arm 4). Hypothesizing, a 2.5-fold increase in the IFN- γ response in the adjuvanted FLU-v arm compared with the non-adjuvanted arm and based on a two-sided type I test with a 95% confidence interval taking into account loss to follow-up of 28% and a 2:1 ratio allocation (antigen vs. placebo), a total of 222 subjects are required to measure significant differences between the treatment arms. For both active arms (arm 1 and 2) and both placebo arms (arm 3 and 4) 74 and 37 subjects are required, respectively.

### Randomization

Each subject screened will be allocated a screening number representing the sequential order in which they are screened. Eligible subjects will be randomized to the four treatment arms. Within each treatment group block randomization will be performed based on the age of the participant (18 to 40 years and 41 to 60 years) to achieve an approximate balanced age distribution [16]. Subjects will be allocated to the next sequential randomization number available at the trial site. Randomization numbers will be generated by the web-based system ALEA [17].

### Blinding

The study will be double-blinded. Preparation of the formulations will be performed by a trained person other than the person vaccinating the subjects. Because an emulsion of Montanide ISA-51 and WFI results in a white liquid formulation, the blinded personnel will only be blinded to the presence of antigen (FLU-v) but not the presence of adjuvant (Montanide ISA-51). The syringes will be labeled with the study number, name of the sponsor, dose, randomization number, date and time of vaccine preparation. The person responsible for the preparation of the formulation will assign each subject to the appropriate treatment group based on the study randomization code. He or she will not reveal the treatment code to the blinded study personnel or perform other study related activities. The pharmacy file including the randomization list, vaccine supplies and all associated documentation will be stored in a locked cabinet within the pharmacy where only non-blinded personnel have granted access.

### Data management

The trial coordinating center (TCC) of the University Medical Center Groningen (The Netherlands) will perform the data management during the trial. TCC will develop an interactive website which will be the major communication instrument to ensure the integration of the program communication and the trial study. Furthermore, TCC will develop electronic case report forms (eCRFs) which will be optimized for scalability in accordance with the Good Clinical Practice (GCP) standards for electronic data entry and flexible export. The eCRFs will be integrated in the interactive website. Data entered into the website will be stored in a database for consistency checks and will be backed up on a daily basis. The database is subjected to secure access control management to allow secure entry, access, analysis and export of data by users and it is also subjected to plausibility and consistency check during the entry process. To ensure personal data protection, all nominal data from subjects will be anonymized.

### Statistical analysis and report

A statistical analysis plan (SAP) will be developed according to the Consolidated Standards of Reporting Trials (CONSORT) 2010 guidelines before the end of the trial [18]. In general, both intention-to-treat (ITT) and per-protocol (PP) analysis will be conducted for the analyses of the CMI and antibody responses, and the reported AEs and SAEs. ITT analysis will include all subjects randomized to receive treatment, irrespective of whether they receive any injections. Subjects who meet the inclusion/exclusion criteria, who comply with the procedures defined in the protocol (i.e. received both vaccinations according to the randomization schedule, provided blood samples for immunogenicity assays on days 0, 42 and 180, not vaccinated with any vaccine or treated with any medication forbidden in the protocol, not with underlying medical conditions forbidden by the protocol) and never have the randomization code broken will be included in the per-protocol analyses. In addition, for safety evaluation all subjects who received at least one injection will be included.

The primary and secondary analysis for specific T-cell responses will be conducted with the PP population using mixed models repeated measures analysis (MMRM), with the baseline level as a covariate. All estimates of treatment differences will be accompanied by 95% confidence interval (CI). For safety, the incidence rate of at least one AE, at least one systemic AE, all solicited AE, any AE, and at least one SAE will be presented with associated 95% CI for each treatment group. SAEs and withdrawals due to AEs will be described in detail. The evaluation of the clinical efficacy will be conducted with the ITT population; for each treatment group the incidence rate of subjects with RT-PCR-confirmed influenza A and/or B will be presented with 95% CI. Scores on a scale of 0 to 3 will be requested for the following symptoms: fever (> 38 °C), malaise, headache, myalgia (muscle and joint point), cough, sore throat, shortness of breath, runny nose, stuffy nose, sneezing and earache. The scores are defined as: 0 = no symptoms, 1 = just noticeable (mild), 2 = bothersome but can still do daily activities (moderate), and 3 = bothersome and cannot do daily activities (severe). The severity of the influenza symptoms among the laboratory-confirmed influenza cases will be summarized in tables of descriptive statistics. In addition, an exploratory analysis will look at the relationship between clinical efficacy and the CMI and humoral responses.

## Discussion

This phase IIb trial is designed to evaluate the immunogenicity and safety of different doses and formulations of FLU-v. A previous phase I trial showed that FLU-v adjuvanted with Montanide ISA-51 elicited cellular immune responses which are important for the protection against influenza illness. In that study the cellular immune response was measured by IFN-γ ELISA. More than a 2-fold increase in IFN-γ was detected in 80 and 100% of subjects who received adjuvanted FLU-v at a low (250 μg) or high dose (500 μg) after 21 days post-vaccination compared to pre-vaccination, respectively [13]. Since the sample size in this study is determined by the earlier trial data obtained with the IFN-γ ELISA assay, this assay is also included as the primary CMI measurement. However, to obtain better quantitative and qualitative insights into the vaccine elicited CMI responses, ICS will also be used to in this phase IIb trial. ICS is an efficient method for simultaneous measurement of antigen specific T cell populations (CD4, CD8) with regard to their putative effector functions (CD107a) and cytokine production capacities (IFN-γ, IL-2, IL-4 and TNFα) [14, 15, 19, 20]. The ICS analysis have been standardized and validated. Aside from the primary CMI assays, additional T cell based assays such as dual ELISpot (IFN-γ and granzyme B) will be performed as an exploratory endpoint since ELISpot has shown to be a sensitive assay to measure influenza-specific CMI [21, 22].

The humoral immune response elicited by FLU-v will be evaluated as a secondary endpoint (total Ig ELISA against the vaccine antigens). Since the vaccine targets internal protein antigens of influenza virus, the vaccine-elicited antibodies are not expected to have neutralizing capacity, but the antibodies could still play a role in activating complement, phagocytosis, or antibody-dependent cellular cytotoxicity (ADCC) that would facilitate killing of influenza-infected cells [23]. In this context, analysis of the Ig subclasses in those serum samples showing high FLU-v specific total Ig titers could provide important information about the putative effector role of FLU-v specific antibodies. Following this, functional assays like ADCC or complement activation could therefore be considered to confirm the role of the FLU-v specific antibodies to combat influenza infected cells. Although FLU-v specific antibodies were low in the previous trial, changes in the vaccine formulation and immunisation schedule are expected to increase the antibody titers in this study.

As an exploratory endpoint the clinical efficacy will be evaluated. This will be determined by the reduction in the incidence of influenza infections and the clinical symptom severity in RT-PCR-confirmed cases during the 2016–2017 influenza season. The symptom scores are determined with a standardised scoring system based on subject-self assessment. This scoring system has been used in a previous phase Ib challenge study in which subjects were challenged with an influenza A virus 21 days after receiving a single dose of 500 μg FLU-v adjuvanted with Montanide ISA-51 or WFI with Montanide ISA-51. In this study investigators found a significant correlation between an increased IFN-γ response and the total symptom score [24]. Although there is not enough power to demonstrate statistical significant clinical efficacy in the current study, there is a chance to investigate immune correlates of protection if the influenza season of 2016–2017 is severe. SEEK is carrying out an influenza challenge study (FLU-v 004) in parallel to this present study. In this challenge study, carried out in collaboration with the National Institute of Health (NIH), subjects will be exposed to H1N1 influenza virus after vaccination. Blood samples will be taken before and after vaccination so that correlation between immune markers and protection can be established. Data from the FLU-v 004 study could be extrapolated to this study, allowing us to identify the number of subjects that have reached a protective immune threshold in this phase IIb study.

FLU-v has been shown to stimulate Th1 responses and the formulation with Montanide ISA-51 enhances this type of response, as seen in previous trials [13, 24]. Vaccine adjuvants are known to increase the response to the vaccine antigens without being antigenic themselves [25, 26]. The immune enhancing effect of ISA-51 is associated with the slow release of the antigen at the immunization site, inflammation and the accumulation of lymphocytes in draining lymph nodes [26, 27]. Montanide ISA-51 has been used in thousands of subjects in clinical trials. Reported adverse events were mainly mild to moderate in intensity and include pain or a reaction at the injection site, headache and myalgia [28, 29]. Montanide ISA-51 creates a water-in-oil emulsion previously shown to enhances the cellular immune responses when added to FLU-v [13, 24]. In fact, the previous phase I trial showed that one administration with non-adjuvanted FLU-v did not induce cellular responses, whereas 80 and 100% of the volunteers receiving 250 μg and 500 μg adjuvanted FLU-v, respectively, showed vaccine specific IFN-γ responses at least 2-fold higher than their pre-vaccination level on day 21 [13]. Emulsions of FLU-v in Montanide ISA-51 and WFI will be prepared by passing the product from one syringe to another locked through a connector (2-syringe mixing) in order to produce stable and homogenous emulsions. The reason for this is that vortex mixing does not give enough shear strength to produce stable emulsions [26]. The majority of AEs reported in the previous FLU-v trials using Montanide ISA-51 were mild. AEs that could be expected in the groups which receive the adjuvanted vaccine include e.g. injection site reactions, pain, erythema and tenderness; however, in order to minimize the AEs the vaccine volume has been halved and the location of the injection has been changed from the forearm to the upper section of the arm.

## Abbreviations

μg: Microgram
AEs: Adverse Events
CCMO: Central Committee on Research Involving Human Subjects
CD: Clusters of differentiation
CI: Confidence interval
CMI: Cell mediated immunity
eCRFs: Electronic case report forms
ELISA: Enzyme-linked immunosorbent assay
GCP: Good Clinical Practice
HA: Hemagglutinin
HI: Hemagglutination inhibition
ICF: Informed consent form
ICS: Intracellular cytokine staining
IFN-γ: Interferon gamma
IL: Interleukin
ITT: Intention-to-treat
M1: Matrix 1
M2: Matrix 2
NA: Neuraminidase
NP: Nucleoprotein
PBMC: Peripheral blood mononuclear cells
PP: Per-protocol
RT-PCR: Reverse transcriptase polymerase chain reaction
SAEs: Serious Adverse Events
SAP: Statistical analytical plan
TCC: Trial coordinating center
TNFα: Tumour necrosis factor

## References

[1] Cox RJ, Brokstad KA, Ogra P (2004). Influenza virus: immunity and vaccination strategies. Comparison of the immune response to inactivated and live, attenuated influenza vaccines. Scand J Immunol.

[2] World Health Organization. Influenza virus infections in humans. 2014. Available at: http://www.who.int/influenza/human_animal_interface/virology_laboratories_and_vaccines/influenza_virus_infections_humans_feb14.pdf. Accessed 13 Apr 2015.

[3] World Health Organization. Influenza (Seasonal) Fact sheet Number 211 March 2014.

[4] Rothberg MB, Haessler SD, Brown RB (2008). Complications of viral influenza. Am J Med.

[5] European Commission. Proposal for a Council Recommendation on seasonal influenza vaccination. 2009. Available at: http://ec.europa.eu/health/ph_threats/com/Influenza/docs/seasonflu_rec2009_en.pdf. Accessed 13 April 2015.

[6] Levy E (1996). French economic evaluations of influenza and influenza vaccination. PharmacoEconomics.

[7] Lee K, Seong BL (1999). Current status for influenza control. Biotechnol Bioprocess Eng.

[8] Skehel JJ, Wiley DC (2000). Receptor binding and membrane fusion in virus entry: the influenza hemagglutinin. Annu Rev Biochem.

[9] Shtyrya YA, Mochalova LV, Bovin NV (2009). Influenza virus neuraminidase: structure and function. Acta Nat.

[10] Verhoeyen M, Fang R, Jou WM, Devos R, Huylebroeck D, Saman E, Fiers W (1980). Antigenic drift between the haemagglutinin of the Hong Kong influenza strains A/Aichi/2/68 and A/Victoria/3/75. Nature.

[11] World Health Organization. Questions and answers. Vaccine effectiveness estimates for seasonal influenza vaccines. 2015. Available at: http://www.who.int/influenza/vaccines/virus/recommendations/201502_qanda_vaccineeffectiveness.pdf. Accessed 13 April 2015.

[12] Berlanda Scorza F, Tsvetnitsky V, Donnelly JJ (2016). Universal influenza vaccines: Shifting to better vaccines. Vaccine.

[13] Pleguezuelos O, Robinson S, Stoloff GA, Caparros-Wanderly W (2012). Synthetic Influenza vaccine (FLU-v) stimulates cell mediated immunity in a double-blind, randomized, placebo-controlled Phase I trial. Vaccine.

[14] Reber A, Katz J (2013). Immunological assessment of influenza vaccines and immune correlates of protection. Expert Rev Vaccines.

[15] Sridhar S, Begom S, Bermingham A, Hoschler K, Adamson W, Carman W (2013). Cellular immune correlates of protection against symptomatic pandemic influenza. Nat Med.

[16] Altman DG, Bland JM (1999). How to randomise. BMJ.

[17] ALEA. Release version 15.1. Available at: https://www.tenalea.com/nkiavl/ALEA/Login.aspx.

[18] Schulz KF, Altman DG, Moher D, CONSORT Group (2010). CONSORT 2010 statement: updated guidelines for reporting parallel group randomised trials. PLoS Med.

[19] Wilkinson TM, Li CK, Chui CS, Huang AK, Perkins M, Liebner JC (2012). Preexisting influenza-specific CD4+ T cells correlate with disease protection against influenza challenge in humans. Nat Med.

[20] Horton H, Thomas EP, Stucky JA, Frank I, Moodie Z, Huang Y (2007). Optimization and validation of an 8-color intracellular cytokine staining (ICS) assay to quantify antigen-specific T cells induced by vaccination. J Immunol Methods.

[21] Salk HM, Haralambieva IH, Ovsyannikova IG, Goergen KM, Poland GA. Granzyme B ELISPOT assay to measure influenza-specific cellular immunity. J Immunol Methods 2013;398-399:44-50. 10.1016/j.jim.2013.09.007 PMC3840047 24055591

[22] Lindemann M, Witzke O, Lutkes P, Fiedler M, Kreuzfelder E, Philipp T (2006). ELISpot assay as a sensitive tool to detect cellular immunity following influenza vaccination in kidney transplant recipients. Clin Immunol.

[23] Jegaskanda S, Job ER, Kramski M, Laurie K, Isitman G, de Rose R (2013). Cross-reactive influenza-specific antibody-dependent cellular cytotoxicity antibodies in the absence of neutralizing antibodies. J Immunol.

[24] Pleguezuelos O, Robinson S, Fernandez A, Stoloff GA, Mann A, Gilbert A (2015). A Synthetic Influenza Virus Vaccine Induces a Cellular Immune Response That Correlates with Reduction in Symptomatology and Virus Shedding in a Randomized Phase Ib Live-Virus Challenge in Humans. Clin Vaccine Immunol.

[25] Wilson-Welder JH, Torres MP, Kipper MJ, Mallapragada SK, Wannemuehler MJ, Narasimhan B (2009). Vaccine adjuvants: current challenges and future approaches. J Pharm Sci.

[26] Aucouturier J, Dupuis L, Deville S, Ascarateil S, Ganne V (2002). Montanide ISA 720 and 51: a new generation of water in oil emulsions as adjuvants for human vaccines. Expert Rev Vaccines.

[27] Aucouturier J, Dupuis L, Ganne V (2001). Adjuvants designed for veterinary and human vaccines. Vaccine.

[28] van Doorn E, Liu H, Huckriede A, Hak E (2016). Safety and tolerability evaluation of the use of Montanide ISA51 as vaccine adjuvant: a systematic review. Hum Vaccin Immunother.

[29] Ascarateil S, Puget A, Koziol M (2015). Safety data of Montanide ISA 51 VG and Montanide ISA 720 VG, two adjuvants dedicated to human therapeutic vaccines. J Immunother Cancer.

